# The dysconnection hypothesis (2016)

**DOI:** 10.1016/j.schres.2016.07.014

**Published:** 2016-10

**Authors:** Karl Friston, Harriet R. Brown, Jakob Siemerkus, Klaas E. Stephan

**Affiliations:** a Wellcome Trust Centre for Neuroimaging, Institute of Neurology, University College London, UK; b Oxford Centre for Human Brain Activity, University of Oxford, UK; c Translational Neuromodeling Unit (TNU), Institute for Biomedical Engineering, University of Zurich and ETH Zurich, Switzerland; d Department of Psychiatry, Psychotherapy and Psychosomatics, Zurich, Switzerland

**Keywords:** Schizophrenia, Dysconnection, Neuromodulation, Bayesian, Predictive coding, Neurogenetics

## Abstract

Twenty years have passed since the dysconnection hypothesis was first proposed (Friston and Frith, 1995; Weinberger, 1993). In that time, neuroscience has witnessed tremendous advances: we now live in a world of non-invasive neuroanatomy, computational neuroimaging and the Bayesian brain. The genomics era has come and gone. Connectomics and large-scale neuroinformatics initiatives are emerging everywhere. So where is the dysconnection hypothesis now? This article considers how the notion of schizophrenia as a dysconnection syndrome has developed – and how it has been enriched by recent advances in clinical neuroscience. In particular, we examine the dysconnection hypothesis in the context of (i) theoretical neurobiology and computational psychiatry; (ii) the empirical insights afforded by neuroimaging and associated connectomics – and (iii) how bottom-up (molecular biology and genetics) and top-down (systems biology) perspectives are converging on the mechanisms and nature of dysconnections in schizophrenia.

## 1 Introduction

The dysconnection hypothesis (Friston and Frith, 1995, Weinberger, 1993) has been implicit from the inception of schizophrenia as a diagnostic construct: for example, Wernicke's *sejunction hypothesis* (Ungvari, 1993) and Bleuler's *disintegration of the psyche* (Bob and Mashour, 2011) provide complementary perspectives on a failure of functional integration in the brain. These early formulations highlight the distinction between an anatomical dysconnection (sejunction hypothesis) and a functional dysconnection (disintegration of the psyche). The dysconnection hypothesis per se is a hypothesis about functional (synaptic) connectivity that is very specific about the pathophysiology; namely, an aberrant modulation of synaptic efficacy. This is potentially important because it speaks to the molecular basis of synaptic gain control in the context of distributed and hierarchical processing in the brain.

The dysconnection hypothesis *precludes* a primary aetiological role for anatomical disconnections. There are simple reasons for this – because schizophrenic signs and symptoms can be elicited by psychomimetic drugs (e.g., NMDAR antagonists), the primary aetiology cannot be attributed to a disruption of white matter tracts – or abnormal neurodevelopmental trajectories. In other words, the fact that psychosis can be induced by simply changing the neuromodulatory status of synaptic integration suggests that the anatomical and neurodevelopmental characteristics of schizophrenia are *consequences* not *causes* of the underlying pathophysiology. Furthermore, while synaptic abnormalities can explain aberrant neurodevelopment (e.g., via activity-dependent pruning), the converse is less obvious. Clearly, this is a rather polemic argument: for instance, ketamine could just produce a phenocopy or the pathophysiology at play may be manifest throughout development, affecting both synaptic function and neurogenesis (Zhang et al., 2016). Perhaps the most important aspect of the dysconnection hypothesis is that it disambiguates between proximal aetiologies at the level of synaptic physiology and neurodevelopmental failures of cell migration and morphogenesis, acknowledging that the two levels contextualize each other. Crucially, if the dysconnection hypothesis can be falsified this would be a great advance – enabling a focus on alternative (e.g., epigenetic) processes (Catts et al., 2013). Having said this, the circumstantial evidence and theoretical support for the dysconnection hypothesis appears to be accumulating as the years pass. In what follows, we summarize some of the key developments.

## 2 The dysconnection hypothesis

The dysconnection hypothesis tries to establish a link between the symptoms and signs of schizophrenia and the underlying molecular and neuronal pathophysiology. Physiologically, it suggests that psychosis is best understood – at a systems level – in terms of aberrant neuromodulation of synaptic efficacy that mediates the (context-sensitive) influence of intrinsic and extrinsic (long-range) connectivity. It proposes that the key pathophysiology lies in the interactions between NMDA receptor function and modulatory neurotransmitter systems. While many (synaptic) theories of schizophrenia postulate a central role for NMDA receptors (Braff et al., 2001, Cull-Candy et al., 2001, Geyer et al., 2001, Javitt and Zukin, 1991, Jentsch and Roth, 1999, Krystal et al., 1994, Olney et al., 1989, Olney et al., 1999), the dysconnection hypothesis highlights the influence of modulatory transmitters on NMDAR-mediated changes in synaptic efficacy. For example, changes in NMDAR conductivity (via phosphorylation), subunit expression and trafficking that follow activation of dopaminergic receptors (Stephan et al., 2009). Crucially, the dysconnection hypothesis explains how the physiological consequences of abnormal modulation of NMDAR-mediated plasticity (such as altered pyramidal cell gain) translate into computational impairments at the level of neuronal circuits – and how this leads to false inference and psychomotor poverty. It is this attempt to close the explanatory gap between pathophysiology and psychopathology that has seen the greatest development over the past years. This development rests on the emergence of the Bayesian brain and predictive coding as formal frameworks for understanding connectivity and computational architectures in the brain:

## 3 The Bayesian brain

There are many computational perspectives that could be called upon to characterize psychopathology. These range from neural network and dynamical systems theory to reinforcement learning and game theory. However, these frameworks do not address the quintessential aspect of schizophrenia; namely, the production of false beliefs. The symptoms and signs of schizophrenia are, almost universally, attended by abnormal beliefs and their behavioral sequelae; for example, paranoid ideation, delusions, hallucinations, and so on. This calls for a theoretical framework that explains *false inference* – and how this false inference is realized neurophysiologically.

A recent paradigm shift in cognitive neuroscience provides exactly the right sort of theory that allows one to talk about false beliefs – and understand how these arise from synaptic pathophysiology. Cognitive neuroscientists now view the brain as a statistical organ that generates hypotheses or fantasies that are tested against sensory evidence. This perspective can be traced back to Helmholtz and the notion of unconscious inference (Helmholtz, 1866/1962). In the past decades this approach has been formalized to cover deep or hierarchical Bayesian inference – about the causes of our sensations – and how these inferences induce beliefs and behavior (Clark, 2013b, Dayan et al., 1995, Friston et al., 2006, Hohwy, 2013, Lee and Mumford, 2003).

### 3.1 Predictive coding and the Bayesian brain

Modern formulations of Helmholtz's ideas usually appeal to theories such as predictive coding (Clark, 2013b, Friston, 2008, Rao and Ballard, 1999, Srinivasan et al., 1982). Predictive coding describes how the brain processes sensory information by optimizing explanations for its sensations: see (Bastos et al., 2012) for a review of canonical microcircuits and hierarchical predictive coding in perception and (Adams et al., 2013a, Shipp et al., 2013) for corresponding treatments of the motor system.

**Fig. 1:**
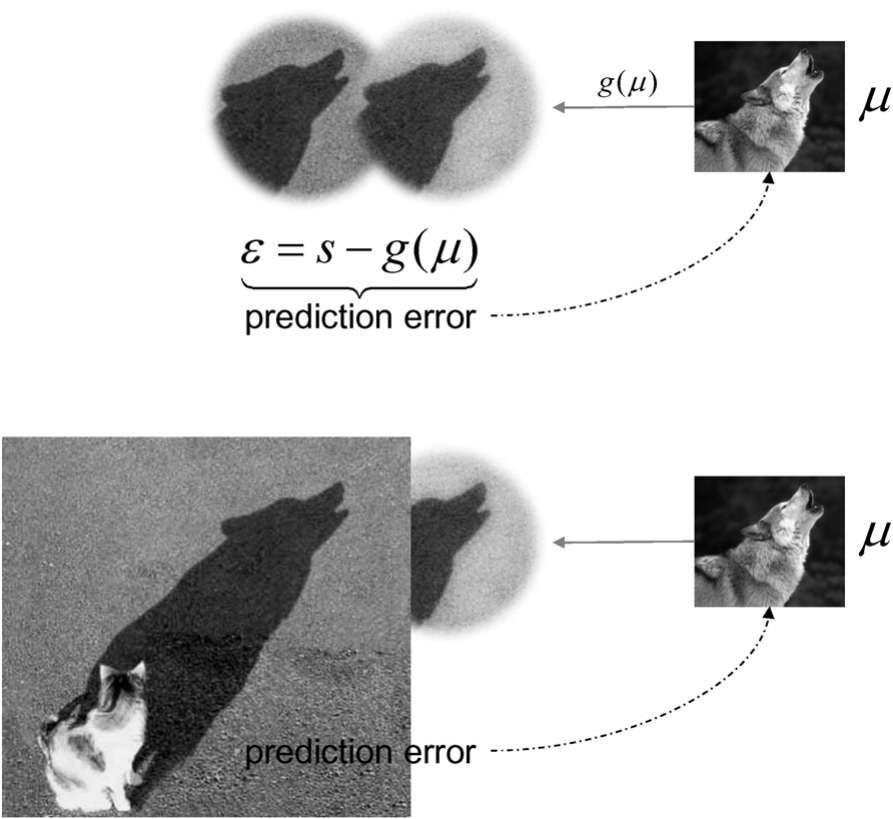
predictive coding deals with the problem of inferring the causes of sparse and ambiguous sensory inputs. This is illustrated in the upper panel in terms of a shadow that can be regarded as a sensory impression. A plausible explanation for this input could be a howling canine. Predictive coding assumes that the brain has a model that generates predictions of sensory input, given a hypothesis or expectation about how that input was caused. Here, the expectation is denoted by *μ* and the sensory prediction it generates is summarized with *g*(*μ*). The prediction error is the difference between the input and predictions of that input. This prediction error is then used to update or revise the expectation, until prediction error is minimized. At this point, the expectation provides the best explanation or inference for the causes of sensations. Note that this inference does not have to be veridical: in the lower panel, the actual cause of sensations was a cat; however, the beholder may never know the true causes – provided that we minimize our prediction errors consistently, our model of the world will be sufficient to infer plausible causes in the outside world that are hidden behind a veil of sensations.

In predictive coding, neuronal representations in higher levels of cortical hierarchies generate predictions of representations in lower levels. These top-down predictions are compared with representations at the lower level to form a prediction error (associated with the activity of superficial pyramidal cells). The ensuing mismatch signal is passed back up the hierarchy, to update higher representations (associated with the activity of deep pyramidal cells). This recursive exchange of signals suppresses prediction error at each and every level to provide a hierarchical explanation for sensory inputs. In computational terms, neuronal activity is thought to encode beliefs about states of the world that cause sensations (e.g., my visual sensations are caused by a *dog*). The simplest encoding corresponds to the expected value or *expectation* of a (hidden) cause. These causes are referred to as *hidden* because they have to be inferred from their sensory consequences. In short, predictive coding represents a biologically plausible scheme for updating beliefs about the world using sensory samples. Fig. 1 tries to convey the basic idea behind predictive coding in terms of minimizing prediction errors.

### 3.2 How precise are our inferences?

Predictive coding provides a compelling (if metaphorical) explanation for many aspects of functional anatomy and perception. However, simply predicting the content of our sensations is only half the story. There is something else that we have to predict; namely, the confidence or *precision* that should be ascribed to ascending prediction errors. Precision is the inverse of variability or uncertainty, and describes the reliability of a signal. Estimating precision speaks to a fundamental aspect of inference in the brain; namely, the encoding of expected uncertainty (Brown et al., 2013, Iglesias et al., 2013, Yu and Dayan, 2005). In other words, not only do we have to infer the *content* of our sensorium but also the *context*, in terms of its (expected or subjective) precision. This represents a subtle but generic problem that the brain must solve, where the solution may rest on modulating the gain or excitability of neuronal populations reporting prediction error (Clark, 2013a, Feldman and Friston, 2010, Friston, 2008).

Heuristically, one can regard the prediction errors that ascend cortical hierarchies as broadcasting ‘newsworthy’ information that has yet to be explained by descending predictions. However, the brain also has to select the channels it listens to. It can do this by adjusting the volume of competing channels. Neurophysiological, this corresponds to adjusting the *gain* of prediction errors that compete to update expectations. Empirical evidence suggests that this boosting or precision-weighting of prediction errors is a central computational process throughout the brain (Iglesias et al., 2013) and may be mediated by neuromodulatory mechanisms of gain control at a synaptic level (Moran et al., 2013).

**Fig. 2:**
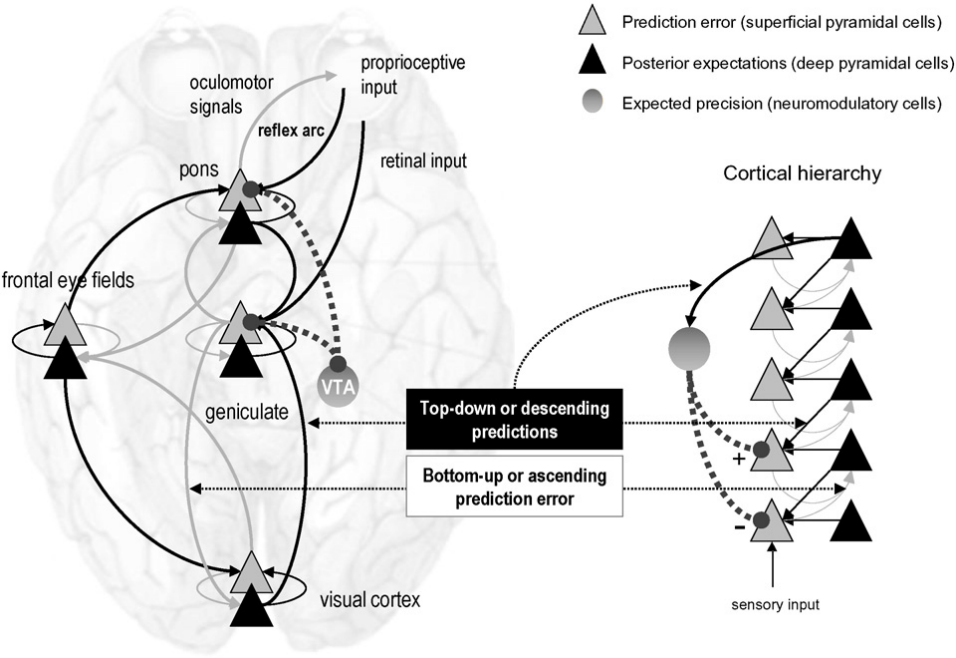
This figure summarizes the neuronal message passing that underlies predictive coding. The basic idea is that neuronal activity encodes *expectations* about the causes of sensory input, where these expectations minimize *prediction error*. Prediction error is the difference between (ascending) sensory input and (descending) predictions of that input. This minimization rests upon recurrent neuronal interactions between different levels of cortical hierarchies. Anatomical and physiological evidence suggests that superficial pyramidal cells (grey triangles) compare the representations (at each level) with top-down predictions from deep pyramidal cells (black triangles) of higher levels. **Right panel**: this schematic shows a simple cortical hierarchy with ascending prediction errors and descending predictions. This graphic includes neuromodulatory gating or gain control (dotted lines) of superficial pyramidal cells that determines their relative influence on deep pyramidal cells encoding expectations (in the same level and the level above). Note that the implicit descending gain control rests on predictions of the precision of prediction errors at lower levels – and can be thought as mediating top-down attentional gain. **Left panel**: this provides a schematic example in the visual system: it shows the putative cells of origin of ascending or forward connections that convey prediction errors (grey arrows) and descending or backward connections that construct predictions (black arrows). The prediction errors are weighted by their expected precision, associated with projections from ventral tegmental area (VTA) and substantia nigra (SN). In this example, the frontal eye fields send predictions to primary visual cortex, which sends predictions to the lateral geniculate body. However, the frontal eye fields also send proprioceptive predictions to pontine nuclei, which are passed to the oculomotor system to cause movement through classical reflexes. Note that every top-down prediction is reciprocated with a bottom-up prediction error to ensure predictions are constrained by sensory information.

Computationally, synaptic gain control therefore corresponds to an encoding of precision, which is reflected in the excitability of neuronal populations reporting prediction errors. This may explain why superficial pyramidal cells (encoding prediction errors) have so many synaptic gain control mechanisms; such as NMDA receptors and classical neuromodulatory receptors like D1 dopamine receptors (Braver et al., 1999, Doya, 2008, Goldman-Rakic et al., 1992, Lidow et al., 1991). Furthermore, it places excitation-inhibition balance in a prime position to mediate ‘precision engineered’ message passing between hierarchical levels (Humphries et al., 2009). This contextual aspect of predictive coding has been associated with attentional gain control in sensory processing (Feldman and Friston, 2010, Jiang et al., 2013) and has been discussed in terms of affordance and action selection (Cisek, 2007, Frank et al., 2007, Friston et al., 2012). Crucially, the delicate balance of precision over hierarchical levels has a profound effect on veridical inference – and may hold the key for a formal understanding of false beliefs in psychopathology (Adams et al., 2013b). Fig. 2 illustrates schematically how neuromodulatory mechanisms may influence hierarchical message passing in the brain.

## 4 Gain control and precision in schizophrenia

So why is the encoding of uncertainty or precision so important for schizophrenia? If schizophrenia is a brain disorder – and the brain is an organ of inference – then its psychopathology must be manifest as a failure of prediction or inference. Analyses of false inference that emerges under failures of predictive coding all point to one abnormality; namely, a failure to properly the encode precision of prediction errors (Corlett et al., 2011, Fletcher and Frith, 2009, Powers Iii et al., 2015, Teufel et al., 2010). There is a growing literature in this area of computational psychiatry that we will briefly summarize. Similar arguments have also been applied in other psychiatric conditions; particularly autism: see (Lawson et al., 2014, Paton et al., 2012, Pellicano and Burr, 2012, Skewes et al., 2014, Van de Cruys et al., 2014).

### 4.1 The psychopathology of perception

The recurring theme in schizophrenia appears to be a *failure to attenuate sensory precision*; in other words, an inability to modulate the gain of sensory prediction errors, relative to higher-level prediction errors that optimize prior beliefs about the causes of the sensory stream. A failure to attenuate sensory precision, from a psychological perspective, means one cannot ignore stimuli and call upon prior beliefs or predictions. In brief, this means that everything is surprising (in statistical terms) because it cannot be predicted. This single computational failure can explain a wide range of signs and symptoms in schizophrenia. For example, if everything is surprising, then it would be difficult to elicit oddball, violation or omission responses – as measured electrophysiologically. This explains impoverished mismatch negativity responses in schizophrenia (Dima et al., 2012, Umbricht and Krljes, 2005, Zarchi et al., 2013). Furthermore, it explains the peculiar resistance to illusions that is characteristic of schizophrenia (Barch et al., 2012, Brown et al., 2013, Butler et al., 2008, Jardri and Deneve, 2013). This resistance can be explained in the straightforward way in terms of a relative attenuation of prior beliefs in relation to the precision of sensory evidence. This follows because illusions rest upon prior expectations to induce a false (illusory) percept. Arguments along these lines provide a nice explanation for perceptual and psychophysical abnormalities in schizophrenia but what about soft neurological signs? Perhaps the most consistent soft sign is a failure of pursuit eye movements (Beedie et al., 2011). Again, when modeled carefully using predictive coding, the characteristic failure of predictive pursuit movements – and paradoxical improvements in the tracking of unpredictable targets – can be reproduced by an imbalance between the precision afforded sensory (visual) prediction errors and prediction errors higher in the cortical hierarchy predicting target motion (Adams et al., 2012).

The above phenomenology provides a fairly comprehensive explanation for many of the trait abnormalities in schizophrenia (i.e., abnormal eye movements, resistance to illusions, suppression of oddball responses etc.). This has led some to suggest that state abnormalities reflect a compensation for overly precise sensory prediction errors. For example, if one reduces sensory precision it is relatively easy to simulate hallucinosis (Adams et al., 2013b). This follows simply from the fact that ascending prediction errors lose their influence over neuronal populations encoding expectations at higher levels in the hierarchy. In other words, perceptual representations are statistically sequestered from sensory constraints (Lawrie et al., 2002). However, this form of hallucinosis does not speak to the positive symptoms of psychosis seen in schizophrenia. To understand the key role of sensory attenuation in explaining delusions we have to turn from perception to action:

### 4.2 The psychopathology of action

To consider agency and action from the perspective of predictive coding, we need to introduce *active inference* (Friston et al., 2011). Active inference is effectively the same as predictive coding but includes the suppression of prediction errors by action or movement. Put simply, there are two ways that we can minimize prediction errors. We can either change predictions (i.e., perception) or we can resample the world to make sensations conform to our predictions (i.e., action). This is nothing more than a description of the motor reflex arc – in which muscles respond reflexively to fulfill top-down predictions of an expected proprioceptive and somatosensory state. However, to engage movement, we have to ignore sensory evidence that suggests we are not moving. This is where the attenuation of sensory precision becomes necessary for action. It is almost self-evident that a failure to attenuate sensory precision will preclude movement – because any prior expectations about moving will be immediately quenched by precise prediction errors correctly reporting the absence of movement.

A corollary of this failure would be a psychomotor poverty not dissimilar to that seen in Parkinson's disease (c.f., bradykinesia and catatonia). This provides a straightforward explanation for some trait abnormalities in schizophrenia; both in terms of psychomotor poverty and in terms of psychophysics. Psychophysically, the attenuation of sensory precision is known as *sensory attenuation* – a reduced sensitivity to the sensory consequences of self-made acts (Brown et al., 2013, Frith et al., 2000). A failure of sensory attenuation is nicely illustrated in the force matching illusion – to which schizophrenic patients are characteristically resistant (Oestreich et al., 2015, Shergill et al., 2005). We can now ask the same question above: what would compensatory increases in the precision of high-level (prior) beliefs look like in a state of psychosis? The answer furnishes a plausible explanation for delusions: to override (unattenuated) sensory prediction errors, it is necessary to increase the precision or confidence in high-level beliefs so that they are more resistant to sensory evidence. The resulting false inference is particularly interesting in the setting of action because there are only two explanations for action: either it was generated internally or by some outside agency. This means that the only explanation for a precise belief about an internally (self) generated act – in the face of precise evidence to the contrary – is that an external agency is preventing that act (or acting antagonistically). Indeed, this is exactly the sort of false expectation (paranoid delusion) that emerges in predictive coding simulations of the force matching illusion, under a failure of sensory attenuation (Brown et al., 2013).

A key insight afforded by this (active inference) account of false inference in schizophrenia is that similar mechanisms underlie hallucinations and delusions: namely, a compensatory increase in the precision of prior (high-level) beliefs, relative to the (unattenuated) precision of (low-level) sensory evidence. This suggests that *delusions are hallucinations about agency or action*. This has an interesting corollary: it means that delusions must necessarily entail false beliefs about behavior, action, agency and intention – of the self or others. Furthermore, rational beliefs formed under irrational precision may necessarily involve outside agencies that are antagonistic, leading to a formal understanding of paranoid ideation.

Computational studies of hallucinations and delusions are still in their infancy; however, they already speak to some fundaments of false inference. Key among these is the relative precision at different levels of the cortical hierarchy. At present, consensus suggests that delusions are associated with unduly precise prior beliefs deep within the hierarchy, leading to recalcitrant explanations for – and attention to – sensory evidence. See (Notredame et al., 2014) for a review of stronger top-down effects in the context of illusions. Furthermore there is an association between top-down effects on perceptual inference and symptom severity, suggesting that “early psychosis and psychosis proneness both entail a basic shift in visual information processing, favoring prior knowledge over incoming sensory evidence” (Teufel et al., 2015).

In summary, to explain false inference in schizophrenia (e.g., delusions and hallucinations), we are drawn to modern versions of Helmholtz's formulation of perception as (unconscious) inference. When deconstructed in terms of neuronally plausible process theories – such as predictive coding – we arrive at the same conclusion that underlies the dysconnection hypothesis; namely, an aberrant modulation of synaptic gain. This convergence equips the dysconnection hypothesis with a formal state theory (hierarchical Bayesian inference) and a process theory (aberrant precision or gain control in predictive coding). The ensuing false inference is explained not in terms of an inability to predict sensory content but a failure to encode the relative confidence that should be placed in sensory evidence, relative to prior beliefs. This can produce a pernicious form of false inference that has been considered at a number of levels; from the genesis of delusions and hallucinations (Corlett et al., 2011, Fletcher and Frith, 2009, Powers Iii et al., 2015, Teufel et al., 2010) through to detailed simulations of hallucinosis, soft neurological signs and characteristic neurophysiological deficits in schizophrenia (Adams et al., 2013b). We now consider the implications for pathophysiology of schizophrenia.

### 4.3 The physiology of gain control

Having a process theory is important because it provides mechanistic predictions that can be tested empirically. Furthermore, it grounds physiological (synaptic) theories of schizophrenia in a functional framework; thereby linking pathophysiological explanations to functional deficits and the patient's beliefs and experiences. Dysfunctional integration at the synaptic level fits comfortably with earlier theories framed in terms of signal-to-noise (Braver et al., 1999, Cohen and Servan-Schreiber, 1992, Winterer and Weinberger, 2004). Furthermore, this putative abnormality is consistent with nearly every synaptic or physiological theory of schizophrenia; ranging from dopaminergic and NMDA receptor dysfunction (Laruelle, 2014, Lisman et al., 2008), GABAergic abnormalities (Gonzalez-Burgos and Lewis, 2012, Lisman, 2012) and a loss of excitation-inhibition balance (Jardri and Denève, 2013). The common theme here is a failure to contextualize the gain of principal or pyramidal cells. This means that any pathophysiology whose downstream effects compromise the modulation of synaptic gain constitutes an ontological class with a common functional expression – a loss of precise inference and subsequent false beliefs about the world (or indeed the self). A focus on synaptic gain control also speaks to the interaction among distinct neuromodulatory mechanisms. An important example here is the interaction between neuromodulation and neuronal oscillations:

One of the most potent physiological mechanisms for synaptic gain rests on synchronous interactions, sometimes referred to as *synchronous gain*. The mechanism here is simple: fast synchronous exchange of neuronal signals increases postsynaptic conductance, synaptic rate constants and postsynaptic gain (Chawla et al., 1999). Almost invariably, this entails interactions between principal cells and inhibitory interneurons (Sohal et al., 2009); thereby linking neuromodulation, abnormalities of fast (e.g., gamma) synchronization and cortical gain control (Spencer et al., 2003, Uhlhaas and Singer, 2010). GABAergic deficits have often implicated parvalbumin positive inhibitory interneurons that target perisomatic regions of pyramidal cells and mediate fast synchronous (gamma) activity. This is important because a number of lines of evidence point to prediction errors being communicated preferentially in the gamma range by superficial pyramidal cells (Bastos et al., 2012).

The gain control implicit in the modulation of inhibitory interneurons underlies several theories of attentional gain: e.g., communication through coherence (Fries et al., 2008). Other examples here include dopaminergic deficits in Parkinson's disease and the modulation of go and no-go pathways – leading to a pathological slowing of neuronal dynamics: e.g., beta activity. Similar themes can be found in schizophrenia, where D2 mediated hyperpolarization in thalamic nuclei may underlie pathological slowing: e.g., delta activity (Belousov and van den Pol, 1997, Ferrarelli and Tononi, 2011).

### 4.4 Summary

To recap, a neurobiological plausible implementation of perceptual inference is predictive coding – a process theory that assigns specific roles to neuronal populations, canonical microcircuits and hierarchical connections (Bastos et al., 2012, Clark, 2013b). The most promising candidate for explaining false inference in schizophrenia is the neuronal encoding of uncertainty (Averbeck et al., 2011). Psychologically, this corresponds to the salience or precision afforded to sensory evidence (Joyce et al., 2013); while physiologically it is thought to be encoded by the gain or excitability of principal (e.g., superficial pyramidal) cells. This resonates with concepts like aberrant salience (Kapur, 2003), while explicitly implicating modulatory neurotransmission in pathophysiology. This line of thinking poses several important questions: for example, how do dopamine and other neuromodulators interact with NMDA receptors and GABAergic interneurons to adjust postsynaptic gain – and is this the key pathophysiological dimension which explains heterogeneity within patients with schizophrenia (Adams et al., 2013b, Stephan et al., 2009). Is the common pathophysiological pathway a failure to optimize excitation-inhibition balance (i.e. gain control) – and is this indexed by aberrations of fast synchronous neuronal activity (Gonzalez-Burgos and Lewis, 2012)? In short, computational and pathophysiological theories of schizophrenia converge on a singular deficit – a failure of neuromodulatory gain control that translates into a failure to contextualize sensory evidence. So what is the empirical evidence for this failure?

## 5 The physiology of dysconnection

**Fig. 3:**
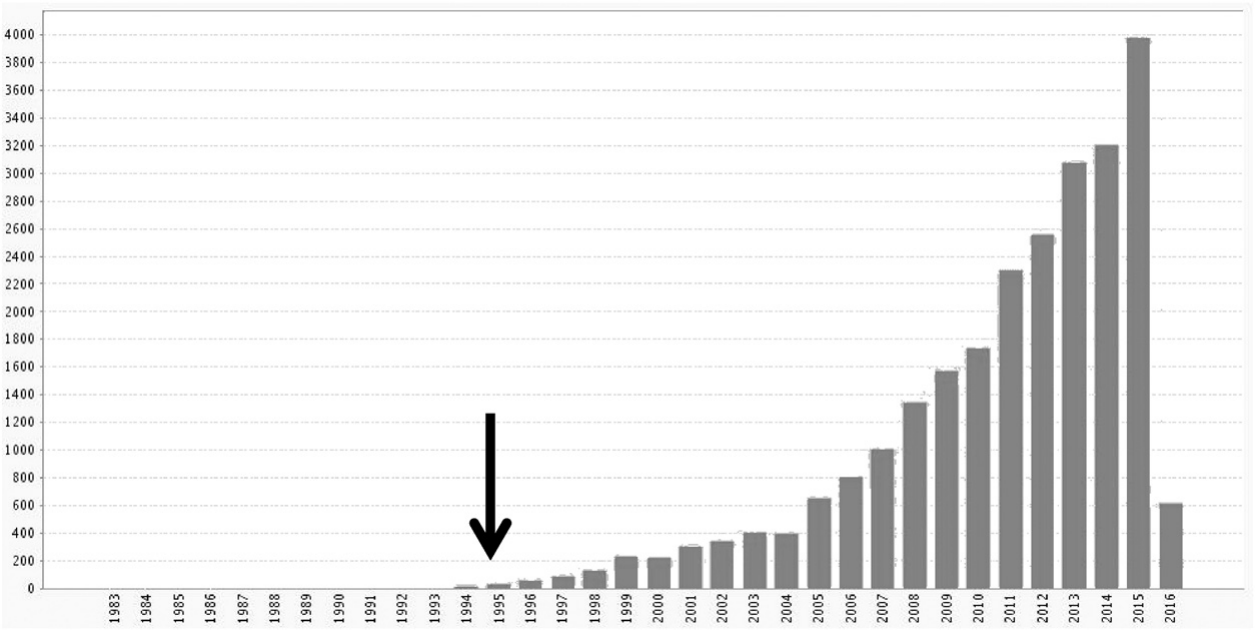
Citations per year, from 1980 to 2016, when searching for TOPIC: (schizophrenia) AND (TOPIC: (disconnection) OR TOPIC: (disconnectivity) OR TOPIC: (dysconnection) OR TOPIC: (dysconnectivity)) in WEB OF SCIENCE™. The arrow indicates the first papers on the disconnection hypothesis were published.

There are many empirical lines of enquiry we could discuss here; ranging from abnormalities in physiological responses to predictability and precision; e.g., the mismatch negativity (Umbricht and Krljes, 2005), to low-level synaptic gain mechanisms in vision (e.g., surround inhibition (Barch et al., 2012)). However, we will focus on systemic (systems-level) measures of connectivity that currently predominate in the neuroimaging literature. The dysconnection paradigm has seen a remarkable surge in interest following the advent of high-resolution neuroimaging – and, in particular, the use of resting state fMRI and EEG activity as a biomarker of dysconnection (see Fig. 3). These studies have looked at functional connectivity in schizophrenia at rest and underlying responses induced by specific tasks: e.g., (Ellison-Wright and Bullmore, 2009, Lawrie et al., 2002, Liang et al., 2006, Lynall et al., 2010, Meyer-Lindenberg et al., 2001, Pettersson-Yeo et al., 2011, Rubinov et al., 2009). The overall picture that emerges from these studies is summarized nicely in (Pettersson-Yeo et al., 2011):

“*In this article*, *we systematically review both the structural and functional connectivity literature in SZ*. *The main trends to emerge are that schizophrenia is associated with connectivity reductions*, *as opposed to increases*, *relative to healthy controls*, *and that this is particularly evident in the connections involving the frontal lobe*. *These two trends appear to apply across all stages of the disorder*, *and to be independent of the neuroimaging methodology employed*.” (but see also (Anticevic et al., 2015) and (Fornito and Bullmore, 2015) for empirical evidence for concurrent reductions and increases of connectivity in schizophrenia).

These studies concern measures of functional connectivity (and associated graph theoretic characterizations), where functional connectivity is defined as the statistical dependence between remote neurophysiological measures. As such, they only provide circumstantial evidence for dysconnection. In other words, they are a compelling ‘smoking gun’. Definitive evidence for systemic dysconnectivity at the synaptic level requires estimates of effective connectivity (defined as the causal influence one neural system over another). In fMRI, estimating effective (directed) connectivity is difficult due to hemodynamic variability across regions; this is usually resolved by biophysically informed state space models like dynamic causal modelling (DCM). Dynamic causal modelling has, in principle, the potential to drill down on very specific synaptic processes implicated in schizophrenia. For example, DRD2 and AKT1 polymorphisms in healthy subjects – implicated in DRD2 signalling – have a selective effect on directed prefrontal-striatal connectivity, while the same polymorphisms alter the dose-response effects of anti-psychotic drugs on cognition in schizophrenia (Tan et al., 2012).

In recent years, DCM studies of schizophrenia have started to appear (Allen et al., 2010, Bastos-Leite et al., 2015, Benetti et al., 2009, Brodersen et al., 2014, Curcic-Blake et al., 2015, Dauvermann et al., 2013, Dima et al., 2012, Kaplan et al., 2016, Schmidt et al., 2014). Almost invariably, these studies disclose abnormal effective connectivity involving the prefrontal cortex. This is consistent with the functional connectivity studies reviewed above. Some DCM studies have looked explicitly for the synaptic correlates of precision. For example, “in processing subsequent information indicating reduced uncertainty of their predictions, patients engaged relatively increased mid-brain activation, driven in part by increased dorsolateral prefrontal cortex to midbrain connectivity” (Kaplan et al., 2016).

These fMRI studies of disconnection are consistent with a failure of top-down modulation (from prefrontal and parietal cortex) of postsynaptic gain or precision in DCM studies of electrophysiological data; especially studies of the mismatch negativity and related paradigms (Dima et al., 2012, Fogelson et al., 2014). But what about structural connectivity?

The dysconnection hypothesis suggests a failure of functional integration in distributed but circumscribed neuronal systems that are particularly dependent upon neuromodulatory afferents (Adams et al., 2013b); e.g., frontal areas in receipt of (mesocorticolimbic) dopaminergic projections. This would be manifest in terms of abnormal functional connectivity as measured with whole brain techniques (Anticevic et al., 2015). Although the dysconnection hypothesis does not call on a form of sejunction hypothesis or leukodystrophy (i.e., it does not posit a disruption of white matter fasciculi), one would not be surprised to find abnormal functional integration producing changes in morphometry – through changes in the composition of the neuropil (Keifer et al., 2015) via trophic effects of NMDARs on dendritic trees and spines (Monfils and Teskey, 2004, Sin et al., 2002) or changes in tractography – through activity-dependent myelination (Fields, 2015). Indeed, anatomical (e.g., cortical thickness) and structural connectivity abnormalities are common in schizophrenia; however, these differences evolve over time (Sun et al., 2016, van Haren et al., 2011), highlighting the importance of (synaptic) plasticity. A meta-analysis of tractography studies of schizophrenia concludes (Ellison-Wright and Bullmore, 2009):

“*Over all studies*, *significant reductions were present in two regions*: *the left frontal deep white matter and the left temporal deep white matter*. *The first region*, *in the left frontal lobe*, *is traversed by white matter tracts interconnecting the frontal lobe*, *thalamus and cingulate gyrus*. *The second region*, *in the temporal lobe*, *is traversed by white matter tracts interconnecting the frontal lobe*, *insula*, *hippocampus*-*amygdala*, *temporal and occipital lobe*. *This suggests that two networks of white matter tracts may be affected in schizophrenia*, *with the potential for* ‘*disconnection*’ *of the gray matter regions which they link*.”

### 5.1 Summary

In summary, the evidence for a systemic dysfunctional integration from non-invasive studies of schizophrenia is overwhelming. Much of this evidence is circumstantial and predicated on measures of functional connectivity; i.e., correlations or coherence among measures of neurophysiology. The consensus of these findings is a functional dysconnection involving prefrontal cortex and key subcortical (e.g., thalamic) and associative cortical (e.g., temporal) nodes. Recent (dynamic causal) modelling of intrinsic (within source) and extrinsic (between source) connectivity suggests a specific failure of intrinsic gain within the prefrontal cortex or descending modulation of synaptic efficacy in hierarchically subordinate structures. So what might mediate these neuromodulatory failures?

## 6 The genetics of schizophrenia

In this section, we revisit the dysconnection hypothesis from the perspective of genetic studies. We review the evidence that the genetic alterations in schizophrenia have pathogenetic implications for NMDA receptors and their interactions with neuromodulatory transmitters. With the discovery of the DISC1 translocation in a large family with high penetrant psychosis in 1990 (St Clair et al., 1990), it was hoped that the genetic architecture of psychosis would quickly emerge. Coarse genetic mapping techniques in the 1990s facilitated the discovery of a number of genes associated with schizophrenia via linkage analysis. The scope of these studies was expanded with the completion of the HapMap project (International HapMap, 2003), enabling the study of large cohorts of disease singletons using single nucleotide polymorphism (SNP) microarray studies. Many large-scale genome wide association studies (GWAS) of thousands of schizophrenia patients and controls have now been performed, identifying many associations between genetic variants and psychosis (Ripke et al., 2013).

**Fig. 4:**
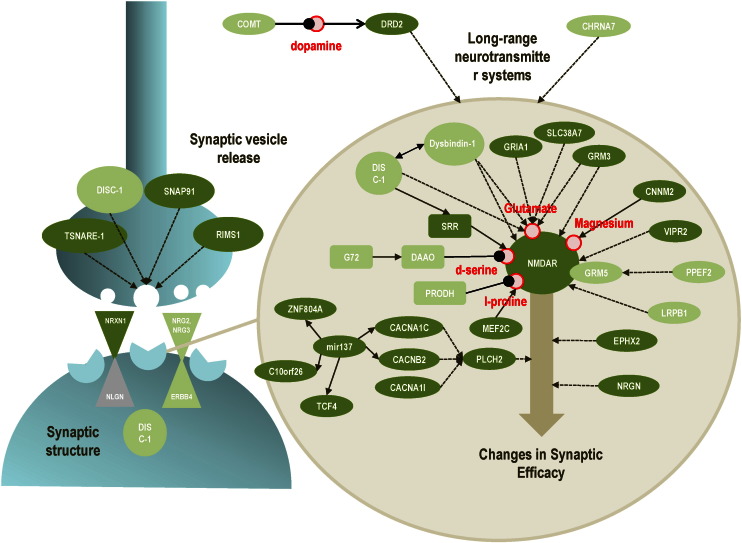
Genes associated with schizophrenia that are implicated in NMDA-receptor function and its interaction with modulatory neurotransmitter systems. Genes with high evidence – genome-wide significance or identified as the relevant gene in a common schizophrenia-associated duplication or deletion – are shown in dark green. Genes with less evidence – replication in two association and/or linkage studies in two different populations, or carrying a rare or de novo deleterious mutation in a patient – are shown in light green. The gene products and functions of these genes are listed in Table 1.

Despite the large number of associations, SNPs only account for up to 23% of the variation in liability for schizophrenia (Lee et al., 2012), with odds ratios of only 1.1–1.25. Additionally, the biological functions of genes uncovered by GWAS are often unknown, with some strongly associated SNPs located in poorly characterized pseudo-genes and zinc finger genes (O'Donovan et al., 2008). However, the largest GWAS study to date, with 36,000 patients and 113,000 controls, recently identified more than 100 SNPs with genome-wide significance (Schizophrenia Working Group of the Psychiatric Genomics, 2014). Notably, genes related to NMDAR function and plasticity at glutamatergic synapses featured prominently (see Fig. 4 and Table 1). These included genes encoding the NR2B subunit (GRIN2A), serine racemase (SRR; which catalyzes the production of d-serine, a co-agonist at NMDA receptors), the GluR1 subunit of AMPA receptors (GRIA1), and various genes encoding calcium channels critical for plasticity at glutamatergic synapses. Further genes identified by this study implicated receptors whose activation is known to modulate NMDAR function, including the D2 receptor (DRD2) and the metabotropic glutamate receptor 3 gene (GRM3).

**Table 1:** Lists of genes that are associated with schizophrenia. A list of specific citations for the table entries is available from the authors. See also (Schizophrenia Working Group of the Psychiatric Genomics, 2014).

I. Gene	II. Evidence	III. Gene product	IV. Known Functions
Genes reported with genome-wide significance, or showing strong evidence for pathogenicity within deletions/duplications			
NMDAR/GRIN2A	GWAS	NMDA receptor subunit	Lynchpin of synaptic plasticity at glutamatergic synapses
GRM3	GWAS	Metabotropic glutamate receptor 3 (mGluR3)	May influence synaptic glutamate levels or NMDA receptor exocytosis
AMPAR/GRIA1A	GWAS	Ionotropic glutamate receptor subunit	Influences postsynaptic glutamate responsiveness.
CACNA1C	GWAS	CaV1.2 voltage-gated calcium channel subunit	Important in NMDA-independent synaptic plasticity in the hippocampus
NRGN	GWAS	Neurogranin – calmodulin-binding protein	Contributes to regulation of post-synaptic calcium levels and long-term potentiation
MIR137	GWAS	mir137 - microRNA	Interferes with transcription of target mRNAs including CACNA1C, DPYD, CSMD1, ZNF804A and TCF4
TCF4	GWAS	Transcription Factor 4	Targets may include other important schizophrenia related genes as well as stress/survival and developmental pathways. Haploinsufficiency causes Pitt-Rivers syndrome, a mental retardation syndrome.
C10orf26	GWAS	Unknown	Unknown
CACNB2, CACNA1I, CACNA1C	GWAS	Voltage-gated calcium channel subunit	Unknown
ZNF804A	GWAS	Zinc Finger Protein transcription factor	Unknown
TSNARE1	GWAS		
EPHX2	GWAS	Epoxide hydrolyse 2	Inhibition reverses PCP (and NMDA antagonist)-induced changes to behavior in mice
SRR	GWAS	Serine racemase	Converts l-serine to d-serine, a co-agonist of NMDA receptors. Mouse SRR knockouts demonstrate NMDA hypofunction
DRD2	GWAS	Dopamine receptors	DRD2 is the main site of antipsychotic action
SLC38A7	GWAS	Glutamine transporter	May be involved in glutamate recycling in the synaptic cleft
PLCH2	GWAS	Intracellular calcium receptor	Involved in calcium signalling during neural development
NRXN1	Deletion, rare mutation	Multiple splice variants yield neurexins – cell-cell adhesion molecules	Involved in the formation, stabilisation and remodelling of both glutamatergic and glycinergic synapses together with NLGN.
MEF2C	GWAS	Transcription factor	Allosteric modulator of the NMDA receptor
CNNM2	GWAS	Cyclin M2	Important in renal regulation of magnesium
VIPR2	Duplication	G-protein-coupled VIPR receptor	VIP known to regulate NMDA receptor activity in the hippocampus.

Genes with weaker evidence of linkage to schizophrenia			
NRG1	Association		NRG1-ErbB4 signalling pathway causes reduction of NMDA currents and long-term plasticity via phosphorylation of NR2B subunit.
ErbB4	Association		
G72	Association	d-amino acid oxidase activator	G72 activates DAOA, which in turn degrades d-serine, a potent co-agonist at the glycine site of the NMDA receptor.
DAAO	Association	d-amino acid oxidase	
DISC1	Association, linkage	Disrupted in schizophrenia-1	Post-synaptic density protein involved in synaptic spine formation, NMDAR trafficking and presynaptic glutamate release. Also stabilises serine racemase.
DTNBP1	Association, linkage	Dysbindin	Post-synaptic density protein which controls NMDAR expression, NMDA-mediated glutamate currents and glutamate release.
COMT	Linkage, deletion	Catechol-*O*-methyltransferase	Degrades dopamine and noradrenaline
CHRNA7	Association, linkage	Acetylcholine receptor	
GRM5	Rare mutation	Metabotropic glutamate receptor 5 (mGluR5)	Physically coupled to NMDAR at the post-synaptic density; co-activation potentiates NMDA currents
PPEF2	Rare mutation	Calmodulin-binding phosphatase	Influences the levels of mGluR5
LRPB1	Rare mutation	LDL-like receptor	Competes for NMDA binding site on PSD-95 structural protein
LRP1	Rare mutation	LDL-like receptor	Competes for NMDA binding site on PSD-95 structural protein
PRODH	Linkage, deletion, rare mutation	Proline dehydrogynase	NMDA receptor agonist at the glutamate binding site

Beyond SNPs, recent advances in genomics have allowed new sorts of genetic variation to be examined. Application of exome capture and sequencing has identified rare, putatively damaging mutations in multiple genes related to NMDAR function in multiplex families (Timms et al., 2013). In addition, trio based study designs (two healthy parents and a mentally ill child) have revealed that schizophrenic patients carry a higher burden of non-synonymous and deleterious de novo SNPs (variants that result from a new mutational event and are unique to the child) than normal controls (Girard et al., 2011, Xu et al., 2011), some of which have been replicated in unrelated patients (Xu et al., 2012). The importance of de novo mutations also fits with the observation that children of older fathers have a two-fold increase in risk for schizophrenia (Kong et al., 2012). Patients with schizophrenia carry a higher number of these de novo mutations than controls (Fromer et al., 2014, Kenny et al., 2014).

Copy number variants (CNVs) have also been investigated in psychosis. For example 25–30% of adult patients with 22q11.2 deletion syndrome (DiGeorge Syndrome), a deletion which includes the *COMT* gene – of relevance for dopamine metabolism (Gothelf et al., 2014) – suffer from schizophrenia (Murphy et al., 1999), making this one of the highest-penetrance genetic changes involved in schizophrenia to date. Other CNVs have also been identified which are significantly associated with schizophrenia (Kirov et al., 2014). Again, a number of duplications and deletions of NMDA-related genes have been discovered to be strongly associated with schizophrenia: e.g., (Rujescu et al., 2009).

These findings have refined our understanding of the genetic architecture of schizophrenia (Fig. 4). Moving beyond the simplistic common-variant-common-disease and rare-variant-common-disease models considered previously, it is now clear that disease susceptibility is most likely due to the interaction of multiple genetic changes – of which a notable subset relates to NMDAR function and its interaction with dopamine. Common variation with low penetrance along with environmental factors may be sufficient to trigger schizophrenia in some patients. Rare high penetrance variants will contribute to the pathology in some patients, possibly leading to severe, early-onset disease (Ahn et al., 2014). In short, the high heritability of schizophrenia is probably caused by alleles that individually are neither necessary nor sufficient to cause disease.

### 6.1 Summary

Recent advances in the genetics of schizophrenia suggest a central role of the NMDA receptor and its interactions with modulatory transmitters in the pathogenesis of schizophrenia. Fig. 4 highlights genes implicated in the modulation of synaptic efficacy that have been associated with schizophrenia. These genes relate to the NMDAR and its interactions with neuromodulatory systems that are, in turn, under control of afferent projections from cortex with NMDAR-dependent plasticity (Bonci and Malenka, 1999); e.g., prefrontal connections targeting midbrain dopamine neurons (Sesack and Carr, 2002).

In terms of the dysconnection hypothesis, the genetic evidence points away from Wernicke's sejunction hypothesis and towards a primary synaptic abnormality: although white matter pathway disruption is an important anatomical finding in schizophrenia, the evidence suggests that developmental failures of axon guidance are unlikely to be the primary aetiology. NMDAR hypofunction disrupts the formation of dendritic spines and growth of dendritic trees (Monfils and Teskey, 2004, Sin et al., 2002), as well as the myelination of axons (Lundgaard et al., 2013). This means that white matter changes can be explained as a consequence of aberrant modulation of synaptic plasticity via NMDA receptors, but not vice versa. This conclusion speaks to the exciting prospect of neurogenetic connectivity studies. Proof of principle is already at hand in, for example, effective connectivity studies of allelic variation in bipolar disorder. “During perception of fearful faces, the presence of the *A* risk [CACNA1C] allele was associated with decreased outflow of information from medial frontal gyrus, which was significantly more marked in patients than in their unaffected relatives and healthy controls” (Radua et al., 2013). Again, we see a selective failure of descending connectivity from the prefrontal cortex.

## 7 Conclusion

Neuroimaging correlates of dysconnection have been shown to be stable over time, heritable and can differentiate between patients and controls (Khadka et al., 2013). These systemic measures of dysconnection may therefore serve as neurobiological indices for defining subgroups of schizophrenic patients. For example, (Brodersen et al., 2014) identified, in an unsupervised way, three distinct subgroups of schizophrenic patients from estimates of prefrontal-parietal-visual connectivity using DCM and a working memory task. Although the search for gold-standard measures of effective connectivity is on-going, there are several well validated phenotypes that depend on intact NMDAR function and neuromodulation. For example, patients with schizophrenia show significantly reduced amplitudes of the mismatch negativity (Umbricht and Krljes, 2005), which can be explained by reduced intrinsic and extrinsic connectivity in the auditory system (Dima et al., 2012, Garrido et al., 2007). Importantly, the MMN is sensitive to manipulations of NMDA and cholinergic receptors (Garrido et al., 2009, Gil-da-Costa et al., 2013). Furthermore, the MMN abnormalities seen in schizophrenia might be attributable to changes in NMDAR-dependent plasticity of forward connections in the auditory system (Schmidt et al., 2013). Interestingly, recent applications of DCM to electrophysiological data suggest that “differential intrinsic recurrent connectivity observed during processing of predictable versus unpredictable targets was markedly attenuated in schizophrenia patients” (Fogelson et al., 2014). Important schizophrenia risk variants are known to influence MMN; for example, a GRM3 variant influences its amplitude (Marenco et al., 2006). This is potentially important, given that mGluR3 receptors regulate NMDAR function (Trepanier et al., 2013). Furthermore, carrying risk variants in the single remaining COMT and PRODH alleles in 22q11 deletion syndrome reduces its amplitude (Zarchi et al., 2013), as with deletion of a NGL1 allele in mice (Ehrlichman et al., 2009). There may be interesting twist to the MMN story schizophrenia: in large cohort studies the reduction in the MMN amplitude shows a large effect size (Light et al., 2015); however, the correlation between the MMN and symptoms is not necessarily high (Wynn et al., 2010). This raises intriguing questions about the underlying trait abnormalities that implicate a failure to modulate synaptic efficacy, relative to the (possibly compensatory) state abnormalities that produce symptoms and signs.

Measures of functional connectivity have also been found to have a genetic basis. A meta-analysis (Mothersill et al., 2012) found that putative schizophrenia risk variants in genes including ZNF804A, PRODH, DISC1 and PPP1R1B, reduced functional connectivity, while the genetic changes examined had no overall effect on structural connectivity. This is interesting given the precedence afforded to synaptic pathology (over axonal pathology) by the dysconnection hypothesis.

### 7.1 Summary

Perhaps the best way to conclude is to think about how we would describe schizophrenia to a patient or relative. On the basis of the above, one could plausibly say:

• At present, our best guess is that schizophrenia is caused by de novo or inherited mutations of one or more genes that influence the expression of (other genes and) proteins mediating the neuromodulation of synaptic efficacy or postsynaptic gain in specific brain systems; particularly prefrontal systems.
• We think that this molecular pathology arises from an abnormal response of the NMDA receptor to specific (e.g. dopaminergic) neuromodulatory receptor activation. This is important because the NMDA receptor mediates activity-dependent changes in postsynaptic gain and subsequent changes in synaptic efficacy.
• The consequences of this abnormal neuromodulation can be diverse; ranging from dendritic and cytoarchitectonic changes, through to activity-dependent changes in myelination, which can be observed microscopically (post-mortem) and macroscopically (using non-invasive neuroimaging).
• The physiological consequences of abnormal gain control are also expressed in terms of a failure to modulate synchronous gain and abnormalities in the coherence of neurophysiological measurements. It is likely that this involves secondary abnormalities in GABAergic neurotransmission that can be summarized as a failure to optimize excitation-inhibition balance or cortical gain control.
• The psychological consequences of failing to modulate synaptic gain can be understood by appreciating that the brain generates hypotheses or beliefs that best explain sensory evidence. A crucial aspect of this process is the encoding of precision or uncertainty that is necessary to select the salient information that updates and contextualizes our beliefs. This attentional selection depends on modulating synaptic gain.
• A failure of neuromodulatory mechanisms that control synaptic efficacy or postsynaptic gain corresponds, functionally, to an inability to augment (attend) or attenuate (ignore) the precision of sensory evidence, relative to the precision of beliefs about the causes of sensory cues. This can lead to false inference (e.g., hallucinations and delusions) that may reflect the brain's attempt to compensate for a pernicious and fundamental attentional failure.
• The particular (prefrontal and dopaminergic) systems implicated in the pathophysiology of schizophrenia may be particularly compromised at later stages of neurodevelopment – or environmental factors that could induce a circular causality (e.g., drug misuse in adolescence).
• A further consequence of false inference is compromised learning that rests on activity-dependent associative plasticity. This means that false inference associated with the positive symptoms of schizophrenia may go hand-in-hand with impaired learning and associated cognitive difficulties.

Clearly, this summary is rather speculative: to close the explanatory gap between pathophysiology at the molecular (synaptic) level and the psychopathology experienced by patients, we still need to identify the links between abnormal synaptic integration, polygenetic predisposition, epigenetics, region-specific gene expression and the implications for hierarchical inference in the brain. Such studies are now starting to appear; e.g., (Mukai et al., 2015, Sigurdsson et al., 2010, Tamura et al., 2016) and one might hope that a complete picture of schizophrenia (or the schizophrenias) will emerge over the next decade or so.

## Role of the funding source

Drs Friston and Brown were supported by the Wellcome Trust (Wellcome Trust grant no 088130/Z/09/Z). Drs Stefan and Siemerkus are supported by the University of Zurich and the René and Susanne Braginsky Foundation.

## Contributors

All authors contributed to the conceptual material described in this review. All authors contributed to the literature review and subsequent synthesis. Authors contributed to and have approved the final manuscript.

## Conflict of interest

All authors declare they have conflicts of interest.
